# Unique Structure and Distinctive Properties of the Ancient and Ubiquitous Gamma-Type Envelope Glycoprotein

**DOI:** 10.3390/v15020274

**Published:** 2023-01-18

**Authors:** Victoria Hogan, Welkin E. Johnson

**Affiliations:** Biology Department, Boston College, Chestnut Hill, MA 02467, USA

**Keywords:** retrovirus, gammaretrovirus, alpharetrovirus, deltaretrovirus, filovirus, reptarenavirus, Env, Gp2, immunosuppressive domain, R-peptide, syncytin

## Abstract

After the onset of the AIDS pandemic, HIV-1 (genus *Lentivirus*) became the predominant model for studying retrovirus Env glycoproteins and their role in entry. However, HIV Env is an inadequate model for understanding entry of viruses in the *Alpharetrovirus*, *Gammaretrovirus* and *Deltaretrovirus* genera. For example, oncogenic model system viruses such as Rous sarcoma virus (RSV, *Alpharetrovirus*), murine leukemia virus (MLV, *Gammaretrovirus*) and human T-cell leukemia viruses (HTLV-I and HTLV-II, *Deltaretrovirus*) encode Envs that are structurally and functionally distinct from HIV Env. We refer to these as Gamma-type Envs. Gamma-type Envs are probably the most widespread retroviral Envs in nature. They are found in exogenous and endogenous retroviruses representing a broad spectrum of vertebrate hosts including amphibians, birds, reptiles, mammals and fish. In endogenous form, gamma-type Envs have been evolutionarily coopted numerous times, most notably as placental syncytins (e.g., human SYNC1 and SYNC2). Remarkably, gamma-type Envs are also found outside of the *Retroviridae*. Gp2 proteins of filoviruses (e.g., Ebolavirus) and snake arenaviruses in the genus *Reptarenavirus* are gamma-type Env homologs, products of ancient recombination events involving viruses of different Baltimore classes. Distinctive hallmarks of gamma-type Envs include a labile disulfide bond linking the surface and transmembrane subunits, a multi-stage attachment and fusion mechanism, a highly conserved (but poorly understood) “immunosuppressive domain”, and activation by the viral protease during virion maturation. Here, we synthesize work from diverse retrovirus model systems to illustrate these distinctive properties and to highlight avenues for further exploration of gamma-type Env structure and function.

## 1. Introduction

**Figure 1:**
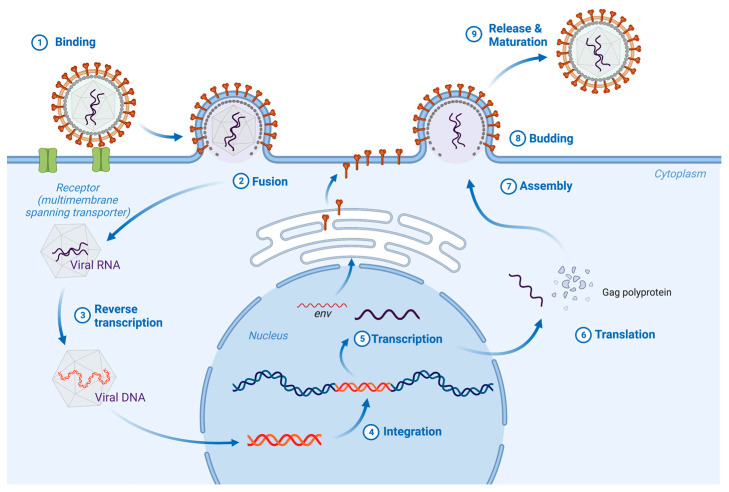
Gammaretroviral Replication Cycle. Schematic of a typical replication cycle for gammaretroviruses.

The family *Retroviridae* comprises two subfamilies, the *Orthoretrovirinae* and the *Spumaretrovirinae* [1]. These are further divided into six and five genera, respectively. For all retroviruses, viral attachment and entry is mediated by the Env glycoproteins, products of the viral *env* gene (Figure 1). Env is expressed as a precursor polyprotein in the secretory pathway. During transit through the secretory pathway, Env is glycosylated and cleaved into two subunits, SU (surface subunit) and TM (transmembrane subunit). Trimers of SU-TM heterodimers assemble into trimeric complexes that are incorporated into the membrane of budding virions. SU contains the primary binding determinants that confer the unique receptor-specificity associated with each virus, while TM mediates fusion of the virion and target cell membranes. For some retroviruses, Env expression also mediates receptor-dependent superinfection interference [2]. The Env proteins of HIV-1 and the related primate lentiviruses have received significant attention from virologists and structural biologists. However, HIV Env comes up short as a model for retroviruses of other genera. For example, viruses of the *Alpharetrovirus*, *Gammaretrovirus* and *Deltaretrovirus* genera share a similar Env protein that is both structurally and functionally distinct from the Env proteins of HIV and the other lentiviruses. Although considerable work has been done on the entry of viruses representing all three of these genera, gammaretrovirus Env proteins are convenient as prototypes both for illustrating the shared features and for highlighting differences between the genera. Therefore, when referring to these collectively we will use the term “gamma-type” Env [3,4]. 

### 1.1. Retroviruses with Gamma-Type Env Proteins

Retroviruses that encode gamma-type Envs include, but are not limited to, the avian leukemia viruses (ALV; *Alpharetrovirus* genus), the murine, feline, and gibbon-ape leukemia viruses (MLV, FeLV, GaLV; *Gammaretrovirus* genus), and the human T-cell leukemia viruses (HTLV-I and HTLV-II; *Deltaretrovirus* genus). Additionally, among viruses in the *Betaretrovirus* genus, a subset known as the “type-D” betaretroviruses are natural recombinants that have acquired gammaretrovirus-related *env* genes, and therefore encode gamma-type Env proteins distinct from those of the canonical betaretroviruses [3,5,6]. Examples of type-D betaretroviruses include Mason-Pfizer monkey virus (MPMV, SRV-3) and squirrel monkey retrovirus (SMRV), among others.

The gamma-type Env can be distinguished from the Envs of lentiviruses, betaretroviruses, and retroviruses of the subfamily *Spumaretrovirinae* by a combination of several criteria, including the presence of an intersubunit disulfide bond between SU and TM, the presence of a C-terminal R-peptide in the immature trimer, two or three highly conserved protein sequence motifs and, in the case of the gammaretroviruses and deltaretroviruses, by a marked bias towards use of multi-pass membrane transporters of the solute carrier (SLC) superfamily as entry receptors [7,8]. Entry mediated by gamma-type Envs also differs from that of other retroviruses in requiring reduction of the intersubunit disulfide bond at a post-binding, but pre-fusion step [9,10,11]. Gammaretroviral entry also differs in multiple aspects from that of influenza A virus (IAV), the textbook example of a class I fusogen [12,13].

### 1.2. Variations on the Gamma-Type Env Are Also Found in Some Non-Retroviruses

The most notable examples of gamma-type Env outside of the *Retroviridae* are the Gp2 proteins of filoviruses (e.g., Ebolavirus, EBOV). Filovirus Gp2 is a distant homolog of the TM subunit of ALV Env [14,15,16]. Reptilian arenaviruses of boid snakes also encode an entry protein with striking sequence similarity to gamma-type Envs [17]. In addition to these RNA viruses, at least one double-stranded DNA virus, Chelonid herpesvirus 5 (ChHV-5), encodes a hypothetical protein (HP35) with significant similarity to a gamma-type Env [4,18]. Given these precedents, it seems likely that additional examples of horizontal gene transfer involving the gamma-type Env, and representatives of other viral taxa remain to be discovered. 

### 1.3. Gamma-Type Env Encoded by Endogenous Retroviruses

Ancient endogenous retrovirus (ERV) loci often contain remnants of retroviral *env* genes, and in some cases, these may retain complete or nearly complete open reading frames with the potential to express functional gamma-type Env proteins [19,20,21,22,23,24,25,26,27]. Gammaretrovirus-related ERV loci are abundant in the genomes of a diverse array of vertebrate species, including mammals, birds/reptiles and amphibians [28,29,30,31,32]. Distantly related ERV loci encoding gamma-type Env can also be found in amphibian genomes [33] and in avian genomes, including recombinant forms [34] and loci closely related to those of exogenous avian leukemia virus (ALV) [29,30]. Mirroring the situation with exogenous betaretroviruses, ERV related to the betaretroviruses include recombinant forms that appear to have acquired a gamma-type *env* gene via recombination [6,34]. Endogenous deltaretrovirus sequences are rare, but a few have been identified in the genomes of a limited number of mammalian species [35,36]; at least one of these has a putative *env* sequence that once encoded a gamma-type Env distantly related to the Env of exogenous bovine leukemia virus (BLV) [37]. Therefore, not only is the gamma-type Env broadly distributed among extant viruses, it is also represented by a majority of the ERV loci found in vertebrate genomes. Collectively, the latter constitutes a “fossil” record of the natural history of the *Retroviridae*, attesting to the ancient origins and prehistoric host-distribution of the gamma-type Env over a period encompassing hundreds of millions of years of vertebrate evolution.

The exogenous epsilonretroviruses of fish include walleye dermal sarcoma virus and the walleye epidermal hyperplasia viruses (WDSV, WEHV; genus *Epsilonretrovirus*). These viruses encode a distinct Env lacking the hallmark features of a gamma-type Env, and gammaretrovirus-related ERV are largely absent from fish genomes [28,38]. However, classification of fish ERVs is based on similarity between the predicted reverse-transcriptase (RT) sequences of the ERV loci and the RT enzymes of exogenous epsilonretroviruses such as WDSV [39]. In contrast, when *env* sequences are used as the basis for phylogenetic comparison, it is evident that many fish ERV arose from viruses that encoded gamma-type Envs [4,40]. Notably, the predicted Env proteins of zebrafish ERV (ZfERV) loci include multiple motifs unique to gamma-type Envs [41]. The number and distribution of fish ERV loci with gamma-type env genes is evidence that related retroviruses once infected the ancestors of modern fish, and raises the possibility that retroviruses with gamma-type Env still circulate in natural fish populations. These observations also indicate that the common ancestor of the epsilonretroviruses employed a gamma-type Env, while the Env encoded by modern exogenous epsilonretroviruses represents a more recent evolutionary development.

### 1.4. Exaptation of ERV-Encoded Gamma-Type Env

ERV loci in vertebrate genomes are also substrates for natural selection, and there are now numerous reports of ERV-encoded proteins that have been coopted by host evolution to serve cellular/organismal functions [19,20,42]. For example, gammaretrovirus-related ERV also include the evolutionarily coopted placental *syncytin* genes of the different mammalian lineages [43]. Other endogenous retroviral genes have been co-opted during host evolution to confer resistance to infection by exogenous retroviruses [44]. In addition to the *syncytin* genes and the receptor interference examples, which are mostly on the order of tens-of-millions of years old, there are numerous examples of ERV loci with intact gamma-type Env ORFs of unknown function [23,27,45,46,47], including some that have remained intact for over 100 million years [22,24,48]. 

#### 1.4.1. Syncytins

The discovery and characterization of the syncytins have been comprehensively reviewed elsewhere [21,43]. Briefly, the syncytins play an instrumental role in placental formation by facilitating cell-cell fusion and formation of the syncytiotrophoblast layer, which is key for the transfer of nutrients from mother to fetus. These include human *Syncytin-1*, which encodes a gamma-type Env derived from the HERV-W family of human ERVs (HERVs), and human *Syncytin-2*, which encodes a gamma-type Env derived from the HERV-FRD family [26,49,50]. Disruption of syncytin expression or function in mice disrupts cell-cell fusion and placental formation, and ultimately leads to termination of the fetus [21]. In addition to the *syncytin* genes of humans and related primates, *syncytin* genes have been identified in many other mammalian lineages as independent captures of retroviral *env* genes. Phylogenetically, most of the mammalian syncytins described thus far cluster with the Env proteins of gammaretroviruses [51,52,53,54].

#### 1.4.2. Retrovirus Envs as Host-Encoded Resistance Genes

When expressed from ERV loci in the host genome, gamma-type Envs may act as barriers to exogenous infection of host tissues by viruses using the same receptor. The murine *Fv4* gene (also known as *Akvr-1*) serves as a well understood example. *Fv4* encodes a gamma-type Env closely related to the Env of ecotropic MLV, and *Fv4* expression confers resistance to ecotropic MLV infection [55,56,57,58,59,60,61]. *Fv4* was first identified in laboratory mice as a resistance locus for Friend MLV [56,57]. Later on, it was identified as an endogenous retrovirus closely related to MLV, containing an intact *env* gene [56,62]. Expression of the *Fv4 env*-like gene in cells has a similar effect to the expression of MLV Env in infected cells: in both cases, resistance to MLV infection occurs, supporting the conclusion that the Fv4 protein creates resistance through the mechanism of receptor interference. Receptor-interference is a common property of gamma-type Envs and is thought to provide a mechanism for superinfection resistance (discussed in a later section). Thus, it is possible that other ERV loci encoding gamma-type Envs evolved to become receptor-specific restriction factors as the result of selective pressure on the host imposed by a related exogenous virus. Indeed, other ERV loci for which there is compelling evidence for a resistance function include the *Rmcf* and *Rmcf2* loci of mice and the *Ev3, Ev6* and *Ev9* loci of chickens [63,64,65,66].

Experimentally, at least, incorporation of Fv4 into MLV virions can also displace or negatively interfere with the function of the wild-type Env [67]. Similarly, Dewannieux et al. demonstrated that Env proteins from gammaretroviruses with different receptor specificities can heteromultimerize and have interfering effects on infectivity in cell culture, raising the possibility that dominant negative interference could be another mechanism by which endogenized Envs act as host restriction factors [68].

The Fv4 protein itself is processed like a normal envelope protein and retains the ability to bind to the mCAT1 receptor (the receptor used by ecotropic MLVs) but has lost its fusogenic potential due to a mutation in the fusion peptide [69]. The Fv4 Env can be incorporated into virions, and has been used in pseudotyping assays with MLV to demonstrate that, though binding occurs, no fusion or entry into the cell can occur [69]. Although difficult to prove, it is tempting to hypothesize that selection as an antiviral defense mechanism would favor ERV Envs that retain their receptor interference functions but lose the ability to mediate membrane fusion or viral entry.

As with many other vertebrates, the human genome contains a multitude of ERV loci, some of which retain some or all of the associated env ORF [25,27,70]. Proving that these have, or once had, antiviral functions is challenging. Among other things, the exogenous forms of the related viruses may have gone extinct millions of years ago. Experimentally, it has been shown that two human ERV-encoded gamma-type Envs (Suppressyn and Syncytin-1/HERV-W) that bind the ASCT2 receptor (SLC1A5) can also induce receptor-interference and render cells resistant to infection by extant retroviruses of other primates that also use ASCT2 as an entry receptor [25,71]. Another strategy is to use ERV sequence information to reconstruct the proteins of an extinct virus, allowing a direct test of the receptor-interference hypothesis. For example, HERV-T loci in the human genome are evidence for an ancient gammaretrovirus that would have infected the lineage leading to modern Old-World primates. Blanco-Melo et al. have identified the receptor that was once used by this virus as MCT1 (SLC16A1) [72]. Similar to Fv4 in mice, the intact *env* ORF of HERV-T in the human genome no longer encodes a fusogenic Env (in this case, due to a mutation in the furin cleavage site between SU and TM). It is, however, still capable of binding to MCT1 and blocking infection by virions carrying a reconstructed version of the ancient, functional HERV-T Env [72]. From this, it is thought likely that the HERV-T Env ORF may have been co-opted early in hominid evolution and preserved by natural selection because its expression conferred resistance to a closely related, exogenous gammaretrovirus. 

### 1.5. Significance

The biological significance of gamma-type Envs is difficult to overestimate. They are associated with diverse viruses, including biomedically relevant viruses such as HTLV-I, HTLV-II and Ebolavirus, they include the mammalian syncytins, which are essential proteins in the development of the placental syncytiotrophoblasts, they are represented by both viral and host proteins, and they are likely to have had a significant impact on the coevolution of retroviruses and their vertebrate hosts. Despite their ubiquity in nature and ancient origins, work on gamma-type Envs for many years has been overshadowed by the focus on HIV-1 and the primate lentiviruses. However, lentiviral Env proteins are imperfect models for understanding gamma-type Envs and the viruses that encode them, and there are no known examples of endogenized, functional lentiviral Envs. In the following sections we attempt to summarize decades of work on retroviruses and ERVs with gamma-type Envs, emphasizing those unique features of gamma-type Envs that distinguish them from the Envs of other retroviruses and highlighting areas for further exploration. 

## 2. Biogenesis

Complete maturation and fusion capability of the Env trimer is achieved through a combination of proteolytic events, including removal of the N-terminal signal peptide (SP) by signal peptidase, cleavage by host-cell furins to release the SU and TM subunits and expose the N-terminal fusion peptide of TM, and cleavage within the virion by the viral protease to remove a short C-terminal peptide (the so-called “R-peptide”) from TM.

The Env precursor protein is synthesized in the rough ER and co-translationally inserted across the membrane. After removal of the signal peptide by signal peptidase, Env travels from the rough ER to the Golgi and trans-Golgi network where it is processed into a trimer with subunits SU and TM. From there the envelope glycoprotein travels to the ER and then finally to the relevant membrane (typically the plasma membrane) [73,74]. Trafficking of gammaretroviral envelope proteins is dictated by specific sequences within the cytoplasmic tail [74,75]. 

Post-translational processing of gamma-type envelope glycoproteins is similar to other retroviral Envs, including glycosylation and furin-mediated cleavage in the secretory pathway. The number of possible glycosylation sites varies across gamma-type Envs with a range of 4–13 attachment sites; a majority of these are located in SU. While glycosylation varies considerably, furin cleavage sites are far more conserved. Cellular furin proteases recognize a specific motif within the envelope protein, typically RRKR, or a basic residue with a dibasic pair (K/R-X-K/R-R) [73,76]. Cleavage by furins leads to separation of the SU and TM subunits and dramatic reorganization of the structure of the envelope protein [77]. This restructuring into the final Env trimer occurs as the glycoprotein precursor travels from the trans-Golgi network to the endoplasmic reticulum, and correct processing by the furin protease has been linked to proper trafficking [73]. Conversely, it has also been reported that furin cleavage mutants result in only a small reduction in infectivity, as long as there are a small number of processed Env proteins available for incorporation [78]. Mutations to the furin cleavage site also lead to a 100-fold reduction of MLV Env’s ability to produce resistance to superinfection [79]. 

## 3. Hallmark Features of Gamma-Type Envs

**Figure 2:**
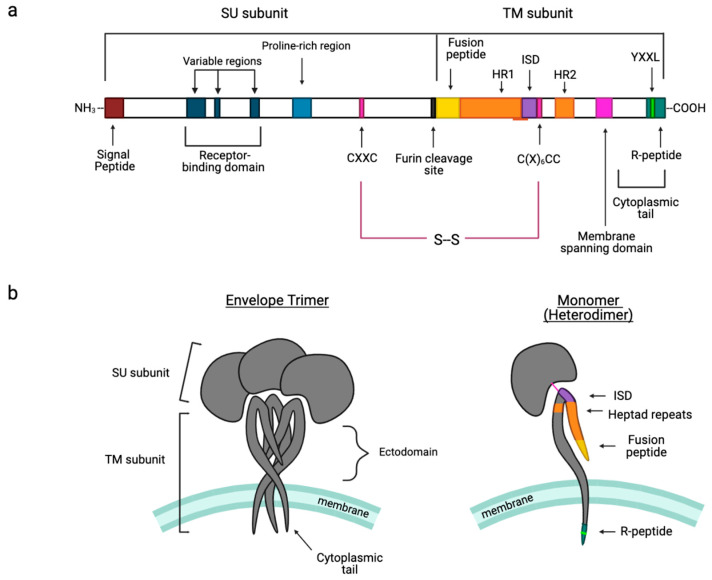
Domains of the gammaretroviral envelope glycoprotein. (**a**) Linear depiction of a gammaretrovirus Env highlighting conserved features. S–S indicates a disulfide bond between the CXXC and C(X)_6_CC motifs. HR1 and HR2 are the two heptad repeat domains of the transmembrane subunit. HR1 overlaps slightly with the immunosuppressive domain (ISD). (**b left**) Schematic of an envelope glycoprotein trimer, composed of three heterodimers comprising SU and TM subunits. (**b right**) Depiction of an Env monomer, highlighting predicted locations of conserved regions including the ISD, HR1 and HR2, the fusion peptide and cytoplasmic tail domains. SU is linked to TM through a disulfide bond, depicted here by a pink line.

Many of the key insights into gamma-type Env structure and function comes from work on murine leukemia virus (MLV) and related gammaretroviruses, while studies of other gammaretroviruses as well as some viruses of other genera, allow for the identification of shared, conserved features and functions that distinguish gamma-type Envs from the Env proteins of HIV-1 and other retroviruses. Below, we highlight some of these, in order of their appearance in the linear protein sequence of MLV Env (Figure 2).

### 3.1. Signal Peptide

The amino terminus of retroviral Env precursors comprises a signal peptide (SP) responsible for targeting the protein to the secretory pathway. SPs also distinguish the gamma-type Env from those of other retroviruses. For example, the SPs of MLV, ALV and HTLV are short (~21–64 residues) and closely resemble canonical SPs of cellular proteins [80]. In contrast, the SPs of lentiviruses (e.g., HIV-1) often overlap with other ORFs, and the SPs of betaretroviruses (e.g., mouse mammary tumor virus (MMTV) and Jaagsiekte sheep retrovirus (JSRV) are longer (>90 residues), multifunctional, and bear little resemblance to cellular SPs [81,82,83,84,85,86].

The Env SP is cleaved off by cellular signal peptidases. Beyond functionality in trafficking and its role as an insertion signal, specific timing of cleavage of the SP may also act as a quality control for properly folded Envs [87]. The addition of cleavable signal peptides has also been shown to enhance packaging efficiency of pseudotyped viral particles [88]. Additionally, the composition of the signal peptide impacts glycosylation, and thus, antigenicity of the envelope glycoprotein [89]. This may indirectly subject the signal peptide sequence to immune related selective pressure. The signal peptide has also been shown to alter levels of incorporation of high mannose carbohydrates into particles which enhances infectivity through increased interactions with certain lectins [90]. 

### 3.2. Receptor Binding Domain (RBD)

The SU subunit of the envelope glycoprotein contains the receptor binding domain (RBD) that gives Env its specificity to a cellular receptor and permits binding of Env which in turn can trigger changes in TM which lead to fusion with the cell membrane. The RBDs of gamma-type Envs, including type-D betaretroviruses and representatives of the *alpharetrovirus*, *gammaretrovirus* and *deltaretrovirus* genera, are located in the amino-terminal half of SU [91,92,93,94,95,96,97,98]. 

The RBD of MLV minimally encompasses a region from amino acid 9 to 230 of the SU [94]. The MLV RBD is probably the best characterized gammaretroviral Env, and at least two variable regions (VRA and VRB) in SU contribute to receptor specificity of MLV Env [99]. Subsequent to coining of the terms VRA and VRB, a third variable region was named VRC. Consequently, in much of the relevant the literature, the N-to-C-terminal order of the variable domains in the linear SU sequence is not alphabetical—VRA, VRC, VRB. More distantly related gammaretroviruses may vary in both the number and sequence of variable regions, but in general these are found in the N-terminal half of SU. Similarly, the receptor binding determinants also map to multiple variable domains in the SU sequences of alpharetroviruses and deltaretroviruses [91,100,101,102,103,104,105,106,107]. Note, however, that while these serve analogous functions as receptor-specificity determinants, the number, location and sequence of variable regions can differ when comparing Env from different genera.

**Figure 3:**
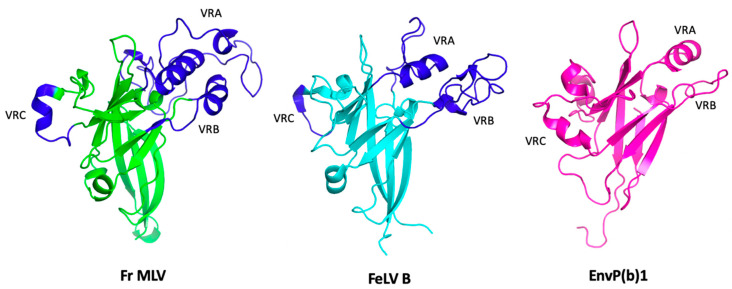
Crystal structures of gammaretrovirus receptor binding domains. Crystal structures of the receptor binding domain (RBD) of Friend MLV (PDB 1A0L), FeLV B (PDB 1LCS) and ENVp(b)1 (PDB 6W5Y). Defined variable regions A, B and C (VRA, VRB, VRC) are shown in blue for MLV and FeLV B. Approximate locations are indicated for EnvP(b)1 based on structurally similar locations (McCarthy et al., 2020). Variable regions are believed to provide receptor specificity among RBDs that recognize different receptors.

High-resolution crystal structures of the receptor-binding domains of two gammaretroviruses (MLV and FeLV) and one endogenous gamma-like ERV have been reported [23,108,109]. Together, the three RBDs represent tens-to-hundreds of millions of years of divergent evolution, yet comparison of the three structures reveals a highly similar core arrangement of beta strands, with the three variable domains arranged on the apical (receptor-facing) surface (Figure 3) [23]. This arrangement is consistent with a role for the variable domains in direct interactions with cell surface receptors.

### 3.3. Proline-Rich Region (PRR)

In gamma-type Envs, the N-terminal domain of SU, including the RBD, is often separated from the C-terminal domain by a proline-rich region (PRR) [95,97,110]. The PRR is not well conserved across divergent gamma-type Envs, indicating that the proline-rich composition may be more important than the specific linear sequence. The PRR is a major antigenic determinant, although antibodies that bind the PRR are non-neutralizing [111]. Structural analysis of the FeLV Env PRR suggested it forms a beta-turn helix [112]. It is thought that the PRR connects the amino terminal half of SU (containing the RBD) to the C-terminal half of SU in a hinge-like way, and that this may be important for activation of TM-mediated fusion [113,114]. Notably, the PRR can tolerate large in-frame insertions, which has proven useful for structure/function studies [115,116,117].

### 3.4. Intersubunit Disulfide Bond and the CXXC(SU) Motifs

**Figure 4:**
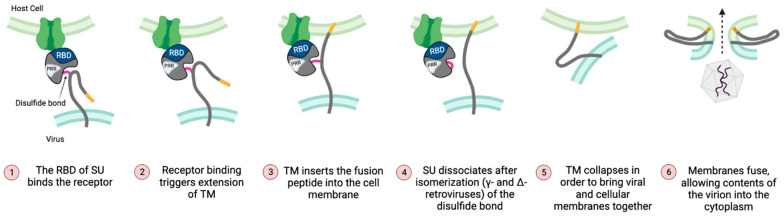
Binding and fusion of a Gamma-type Env protein with a host cell membrane. Env is shown as a single heterodimer rather than a trimer for simplicity. The receptor binding domain (RBD) is shown in blue, and the proline rich region (PRR) is shown in white. After binding of the SU subunit to a receptor, isomerization of the disulfide bond occurs, resulting in conversion to an intra-subunit bond within the CXXC motif of the SU subunit, releasing the SU subunit and ultimately resulting in fusion mediated by the TM subunit. The conformational change that TM undergoes is irreversible, and TM cannot revert to carry out fusion again.

In contrast to the Env complexes of lentiviruses (e.g., HIV-1) and betaretroviruses (e.g., MMTV), the SU and TM subunits of gamma-type Envs are covalently linked via a labile disulfide bond that is crucial for proper Env stability and plays a role in the conversion of the receptor bound Env complex to a fusion-active form [10,11,118,119]. For gammaretroviruses, deltaretroviruses, and type-D betaretroviruses, the bond forms between a CXXC motif in SU and a highly conserved CX_n_CC motif in TM (typically, n = 6 residues) (Figure 2). Isomerization results in loss of the inter-subunit bond and formation of an intra-subunit bond between the cysteines of the CXXC motif, followed by dissociation of SU and refolding of TM into the hairpin, post-fusion conformation [120,121] (Figure 4). 

**Figure 5:**
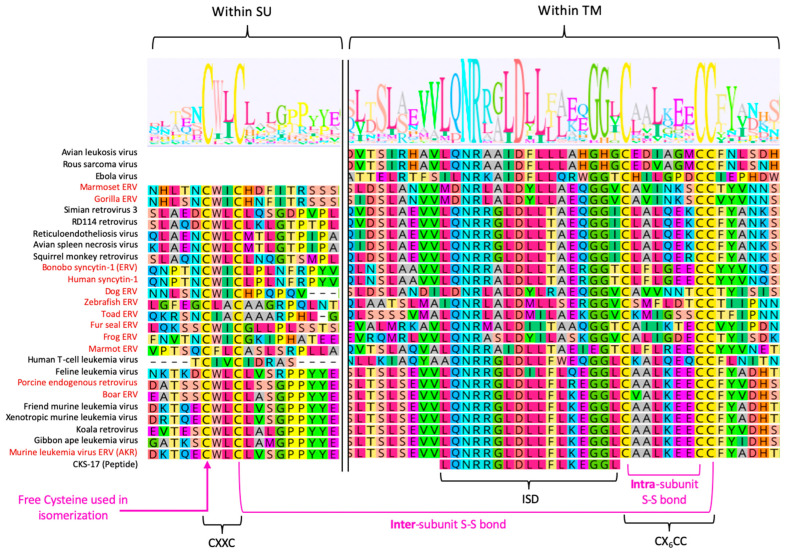
Conservation of CXXC, ISD and CX_6_CC motifs of gamma-type Envs. Geneious amino acid alignment of 27 gammaretroviral and gamma-like Env sequences and the CKS-17 peptide. Endogenous sequences are shown in red while exogenous viruses are shown in black. On the left is a section from SU containing the CXXC motif (which is absent from alpharetroviral and filoviral entry proteins). The CXXC contributes to an intersubunit disulfide bond with the third cysteine in the CX_6_CC motif in TM. On the right is a section of sequence from TM that contains the highly conserved region of the immunosuppressive domain (ISD)**.** The CKS-17 peptide was used to originally define the region of the ISD; also highlighted is the intra-subunit bond that forms within the CX_6_CC motif. RSV and ALV are classified as alpharetroviruses, SRV3 is a betaretrovirus with a gamma-like Env, and HTLV is a deltaretrovirus; also notable is the gamma-like glycoprotein of Ebola virus which is a member of Filoviridae. Accession #s in order of figure: AFV99542.1; BAD98245.1; Q05320.1; ACI62863.1; AGI61275.1; P07575.1; ABS71857.1; QXV86750.1; P31796.1; NP_041262.1; NP_001291475.1; Q9UQF0.1; XP_022270294.1; AAM34209.1; XP_040289417.1; XP_025743503.1; QXP50143.1; XP_015345157.1; AAAU04934.1; AYG96595.1; ACD35952.1; AAQ88184.1; P26804.1; AEI59727.1; ALX81658.1; ALV83307.1; AAB03092.1.

The CXXC sequence is typically found within the C-terminal half of SU of gammaretroviruses, type-D betaretroviruses [122] and deltaretroviruses [107]. However, the location can vary, and in some gamma-type Env it may be located N-terminal to the RBD (e.g., SYNC2). The two internal positions display a bias towards hydrophobic residues, intriguingly similar to the active sites of disulfide exchange enzymes (Figure 5) [11]. The SU of alpharetrovirus Env proteins lack a CXXC motif, and instead the intersubunit bond forms between a cysteine in SU and the CX_6_CC motif in TM (see later subsection). ALV SU contain an unpaired cysteine, but surprisingly, this does not seem to be involved in isomerization or reduction of the intersubunit bond [123]. As with gammaretroviruses, binding of ALV SU to a receptor also triggers extension of TM and insertion of the fusion loop; however, in the case of ALV Envs, completion of fusion requires endocytosis and a concomitant drop in pH [100,124,125].

### 3.5. Fusion Peptide

Retrovirus Envs are class I fusion proteins, with fusion peptides (FP) located at or near the N-terminus of TM [126,127]. Gamma-type Env can have N-terminal FP, as is found in most gammaretroviruses and deltaretroviruses, or the FP can comprise an internal “loop” near the N-terminus of TM demarcated by disulfide bonded cysteines, as is the case for alpharetroviruses [100]. As with other class I fusion proteins, retroviral TM initiates fusion through insertion of the fusion peptide into the host cell membrane, after which refolding of TM brings the cell and viral membranes together to form a fusion pore and allow release of the contents of the virion into the host cell (Figure 4) [126]. The fusion peptide comprises primarily hydrophobic residues, although in the case of Moloney MLV (MoMLV) this hydrophobic region is followed by a glycine and threonine-rich region which may also be important for proper fusion [128]. Mutational analyses of varying retroviral Envs have shown that mutations within the fusion peptide often reduce fusogenicity and overall stability of the Env trimer [128,129]. When the fusion peptide corresponds to the N-terminus of TM it overlaps with the furin cleavage site, which is crucial for proper processing of the envelope protein into its two subunits. Mutations to this region have also been shown to result in incomplete processing, in addition to loss of fusion [128]. The general structure of the fusion peptide is conserved across Envs and commonly includes an ordered region, such as a 𝛽-sheet, followed by a turn or loop, followed by another ordered region such as an 𝛼-helix [130,131]. The peptide is capable of flipping between ⍺-helix and 𝛽-sheet conformations depending on the environment [130]. 

### 3.6. Heptad Repeats

In type I fusion proteins, including retroviral envelope glycoproteins, coiled-coil ⍺-helices formed from heptad repeats lie adjacent to regions directly involved in fusion [132]. In retroviral envelope proteins these comprise two heptad repeat regions (HR1 and HR2) in the ectodomain of the TM subunit. The first heptad repeat sequence, HR1, is just downstream of the fusion peptide, and is also referred to as the N-helix or N-heptad. Triggering of the fusion mechanism induces a series of conformational changes through which the three HR2 peptides wind up anti-parallel to the three internal HR1 regions in the form of a tightly packed six-helix bundle that represents the post-fusion conformation of TM [133,134]. The hairpin turn linking these is sometimes referred to as the “chain-reversal” region. Notably, the MLV HR2 (the C-helix) is significantly shorter than the corresponding C-helix of HIV [135]. Interestingly, peptides derived from the HR1 region (the N-helix) are capable of inhibiting fusion, possibly through inducing a post-fusion conformation and thus rendering the Env non-fusogenic [133,135]. 

Comparison of gamma-type Envs, including representatives of different genera and endogenous sequences found in a diverse range of vertebrate genomes, revealed that many non-mammalian gamma-type Envs share an N-glycan attachment site in or near HR1 [3,24]. Notably, the glycan attachment site is also found in Gp2 of filoviruses and certain snake arenaviruses [3,17,24,136,137,138]. The presence of a glycan attachment motif in this region of type 1 viral fusion proteins introduces a “heptad stutter” that disrupts the periodic 3-4-3-4 spacing of hydrophobic residues that define heptad repeats, and consequently has the potential to influence interhelical interactions, such as those that are involved in entry and fusion. Whether the presence or absence of the glycan (or the attachment motif) results in structural or functional differences between non-mammalian and mammalian gamma-type Envs remains to be determined.

### 3.7. Immunosuppressive Domain (ISD) and the CX_6_CC Motif

Gamma-type Env sequences are easily distinguished from those of other retroviruses by a stretch of ~26 highly conserved residues between HR1 and HR2 (Figure 5). This sequence comprises the most conserved, continuous stretch of sequence in gamma-type Envs, facilitating alignments and phylogenetic comparisons of highly divergent viruses [16]. The first ~17 residues comprise a motif commonly referred to as the immunosuppressive domain, or ISD. The next nine residues comprise the previously described CX_6_CC motif (see previous section). Two of the three cysteines form a conserved intrasubunit disulfide that is also found in TM subunits of non-gamma-type retroviruses, including betaretroviruses and lentiviruses. In contrast, the third cysteine is unique to the TM subunits of gamma-type Envs and is involved in forming the hallmark intersubunit disulfide bond with SU. Together, the combined ISD and CX_6_CC sequence comprises a telltale signature for reliable identification and classification of retroviruses and ERV that encode gamma-type Env proteins [3,119].

The ISD is so-named because a synthetic peptide corresponding to 17 residues immediately upstream of the CX_6_CC element of MLV was observed to have immunosuppressive properties [139,140]. The original peptide, CKS-17, was shown to have a variety of immunosuppressive effects on lymphocyte populations [139,140,141,142]. Later studies reported that the full envelope proteins of MLV and MPMV are capable of inhibiting tumor rejection in mice [143,144]. It has been proposed that the ISD of the placental syncytins also exerts immunosuppressive effects that contribute to maternal tolerance of foreign antigens expressed by the developing fetus, and the same tumor rejection model was used to test the immunosuppressive properties of the syncytin proteins. Intriguingly, it was found that Syncytin-1 showed no immunosuppression, which led to the isolation of two residues within the ISD that appear to switch immunosuppressive effects on and off [145]. While the immunotolerance hypothesis is intriguing, confirming that the results of cell-culture and murine models accurately reflect the in vivo function(s) of syncytins continues to be an open question, and a very difficult one to answer. 

When similar amino acid mutations were introduced into the MPMV and MLV envelope proteins, inhibition of tumor rejection was lost, possibly reflecting a loss of immunosuppressive activity [145]. Wild type and ISD mutant MLV produced similar levels of infection in immunocompromised mice, while in immune competent mice the putative loss of immunosuppressive properties of Env correlated with reduced infectivity [146]. Together, these results suggest that the immunosuppressive functions of the ISD can be genetically separated from pleiotropic effects on viral entry. However, Eksmond et al. found that there was in fact a severe effect of the double mutant on MLV infectivity, but that the defect depended on expression of the cognate receptor in the producer cell [147]. In contrast, when the mutant virus was produced in human cells (lacking the receptor), there was little or no effect of the mutations on infectivity. Interestingly, when the receptor was overexpressed in human cells, infectivity of the wild-type virus was also affected. These observations highlight the difficulties in separating the proposed immunosuppressive effects from effects on the entry functions of Env. One intriguing possibility is that the conservation of the ISD belies a mechanism for stabilizing Env during biogenesis in receptor-expressing cells [147].

To date, there is no unifying molecular mechanism that satisfactorily explains all of the various experimental phenotypes attributed to the ISD. It is particularly challenging to conceive of a hypothetical immunosuppressive mechanism that would be operational in hosts as divergent as mammals, fish, birds/reptiles and amphibians, let alone one that would involve viruses representing a wide range of tissue tropism and disease manifestations and mediate immune tolerance of fetal antigens during pregnancy in mammals. One possibility is that the immunosuppressive effects are unique to a subset of mammalian retroviruses; however, the broad conservation of the ISD sequence among non-mammalian viruses still needs to be explained. Alternatively, the high degree of conservation of the ISD sequence could be an indication that it forms a critical structure or provides an essential function related to viral entry. Indeed, mutations of the ISD that result in defects in infectivity or replication in cell culture are consistent with the possibility that the ISD has a fundamental role in viral replication [147,148]. Such a structure or function could be independent of, or in addition to, a secondary immunosuppressive effect. What those structures/functions might be remains to be discovered. The invariant co-occurrence of the adjacent ISD and the CX_6_CC motifs in all gamma-type Envs hints at one intriguing possibility: perhaps the ISD motif contributes to formation or stabilization of the hallmark disulfide bond between TM and SU. This would not preclude an additional immunosuppressive function, but would necessarily complicate experiments aimed at disentangling putative immunosuppressive functions from the well-established contributions of Env to virion assembly, attachment, fusion, entry and receptor-interference. 

### 3.8. Cytoplasmic Tail

The cytosolic C-terminal domain of TM, or cytoplasmic tail (CT), begins just after the hydrophobic membrane-spanning segment. TM retains this topology in the virion, with the ectodomain on the outside of the viral membrane and the CT oriented towards the interior of the virion where it can interact with the viral structural proteins and the viral protease. The CT varies in length considerably between retroviruses; lentiviruses, such as HIV, have CTs over 100 residues long. Gammaretroviral envelope proteins, by contrast, typically have short cytoplasmic tails of around 25–35 residues [74]. CT sequences are highly variable and difficult to align, but often contain similar motifs that are thought to function in proper trafficking, cellular localization, and viral assembly [149]. For both the MLV and MPMV Envs, the cytoplasmic tails contain two conserved motifs, a dileucine motif and a tyrosine motif. The dileucine motif directs trafficking of Env from the TGN to endosomal compartments. These Envs then appear to traffic back to the TGN, which is controlled by the tyrosine motif. Mutations in both of motifs cause mis-localization of the envelope protein [150]. Additionally, both motifs may be involved in recruiting clathrin adapter protein complexes although the exact mechanisms and cellular factors involved in correct trafficking are still unknown [151]. Gamma-type Env CTs also often contain at least one YXXΦ motif that is thought to be essential for Env incorporation in virions [152]. 

Beyond trafficking, there is evidence that the cytoplasmic tail is involved in direct interactions with matrix (MA) and is crucial for incorporation of Env into particles [153]. Consistent with this observation, it has been shown that the CT determines pseudotyping compatibility and viral core preference by envelope proteins [154]. When Envs were expressed in cells in the absence of the other viral structural proteins, they were found to be initially randomly distributed across the plasma membrane. However, once viral core proteins were also expressed, Env trimers were found non-randomly clustered at viral budding sites. This demonstrated that the envelope proteins were actively recruited to sites of assembly, and additionally showed preference for certain viral cores over others that was dictated by the cytoplasmic tail [154]. Similarly, work in HIV has also shown that sites of virion release at the cell surface are determined by the envelope glycoprotein.

### 3.9. R-Peptide

Another distinguishing feature of gamma-type Envs is the presence of a so-called “R-peptide”, comprising the C-terminal most residues of the CT. The R-peptide must be cleaved off during virion maturation to activate the fusogenic potential of the Env complex [155,156,157,158,159,160]. The addition of the R-peptide of MLV to heterologous entry proteins, including divergent ones such as influenza HA, is sufficient to inhibit fusion [161]. However, incompatibility between a viral protease and the R-peptide cleavage site can reduce infectivity of virions pseudotyped with heterologous Env proteins [162]. It is interesting to speculate that such an incompatibility could limit the acquisition of new env genes by recombination between divergent retroviruses. For an interesting perspective on the origins of the term “R-peptide” see Rein, 2021 [163].

The MLV Env R-peptide comprises the C-terminal 16 amino acids of the cytoplasmic tail [156,164,165]. The viral protease cleaves the R-peptide during assembly and maturation of the viral particle. Cleavage of the R-peptide primes the transmembrane subunit for fusion, as MLV particles with an Env that contain an R-peptide, or are incapable of cleaving the R-peptide, do not induce cell-cell fusion [165]. Experimental cleavage or truncation of the R-peptide is sufficient to restore fusogenicity and infectivity to the MLV Env [165]. The domain containing the R-peptide forms a coiled coil prior to cleavage and fusion, and it is thought that this conformation blocks isomerization of the disulfide bond between SU and TM, or keeps Env in a non-fusogenic conformation [166,167]. However, the R-peptide does not appear to be a completely independent inhibitory mechanism, as mutations upstream in the cytoplasmic tail can reduce the inhibitory effects [168]. 

Interestingly, in addition to control of fusion, MLV envelope proteins with an R-peptide bind to the mCAT1 receptor less efficiently than envelope proteins without an R-peptide, and truncation of the R-peptide results in reduced incorporation of envelope protein into virions [169]. When the R-peptide was removed from a gammaretroviral Env it spread poorly in cell culture, but given time it acquired a compensatory extension of the C-terminus. This also restored the colocalization between the envelope protein and Gag, implying an additional role for the R-peptide in assembly of viral particles [170]. The R-peptide of MLV, and the entire cytoplasmic tail, is dispensable for assembly with HIV particles, consistent with the fact that HIV glycoproteins do not contain an R-peptide. However, the presence of the MLV R-peptide provides specificity for MLV Gag particles over HIV Gag [162]. This role in assembly is still poorly understood, as are the precise mechanisms through which the R-peptide is able to inhibit fusion.

## 4. Receptor Interactions of Gamma-Type Envs

The gamma-type Env complex engages with a cognate receptor at two distinct stages in the viral life cycle. First, as with all retroviruses, receptor-binding by mature gamma-type Env present on the virion initiates the cascade of events leading to fusion and viral entry into a target cell. Second, interactions between the immature gamma-type Env and the viral receptor can occur within the infected, virus-producing cell, resulting in reduced susceptibility of the cell to re-infection—a phenomenon known as receptor interference or superinfection resistance. As described in a previous section, the immature, cellular form of Env is distinguished from the mature, virion-associated form by the presence of a C-terminal R-peptide. One likely function of the R-peptide is to prevent these intracellular interactions (i.e., the interactions that result in receptor-interference) from premature triggering of fusion. Similarly, the R-peptide can suppress premature fusion between the producer cell and adjacent target cells. Only those Env molecules that successfully traffic to sites of assembly and are incorporated into nascent virions are activated by the viral protease, ensuring that Env-receptor interactions involving the mature virion and a target cell initiate the fusion and entry process. 

### 4.1. The RBD Regulates Both Initiation and Completion of Fusion

For both ALV and MLV, receptor binding results in extension of TM and insertion of the fusion peptide into the target cell membrane [121,133,171]. However, a second trigger is required before TM can refold into the hairpin structure typically associated with completion of fusion [124,125]. For ALV Env proteins, this occurs when low pH is encountered during viral entry via the endosome [124,172]. By contrast, for MLV (and other gammaretroviruses), isomerization of the intersubunit disulfide bond between SU and TM is necessary for refolding of TM and completion of fusion [120,121].

The pH-dependence for ALV Env is well established [124,172]. By comparison, entry by amphotropic and xenotropic MLV have repeatedly been shown to be pH-independent, demonstrating no defect in fusion or infection when treated in ways that raise pH and prevent acidification of endosomes [173,174]. Interestingly, lower pH within endosomes has been suggested to play a role in fusion for ecotropic MLV, with pH dependence relying on c-terminal residues of SU [173]. However, it has also been shown that once the R-peptide of ecotropic MLV Env is cleaved off, low pH is no longer required for fusion [159]. This suggests that pH-dependence for ecotropic MLV may not be connected to fusion, and may be important after membrane fusion has occurred [159]. Whether or not pH plays a role in entry, and the underlying molecular basis for pH-dependence when it is observed, may differ even among related viruses. Given the natural host distribution and extreme diversity of gamma-type Envs, the effects of pH cannot be assumed based on taxonomic relationships, but rather must be determined experimentally for any given virus.

Multiple, independent lines of evidence point towards involvement of gammaretrovirus RBDs in a second, post-binding step that leads to isomerization of the intersubunit disulfide bond and refolding of TM into the hairpin structure associated with completion of fusion. For example, receptor binding and fusion can be uncoupled by mutation of a key histidine at the N-terminus of the MLV RBD. Virions containing the ΔHis mutant Env bind to the cognate receptor, but do not complete the fusion process [94]. The mutant can be rescued by providing a soluble RBD (sRBD) in-trans, and it is thought that rescue is evidence for a second interaction between the RBD and the C-terminal domain of SU [175,176,177]. Similar observations have been reported for gibbon ape leukemia virus (GaLV) [178]. The phenomenon also extends to the T strain of feline leukemia virus (FeLV-T). FeLV-T virions can bind to target cells, but entry depends on transactivation by a naturally occurring, secreted RBD encoded by a defective FeLV-B related ERV locus [108,179,180,181]. In some cases, rescue in-trans can be achieved using combinations of Envs with sRBDs derived from related viruses with different receptor-specificities; however, the target cells must express the cognate receptor for the transacting sRBD [12,176].

These observations led to a model comprising two sequential interactions involving an RBD [12]. First, an RBD binds to a cognate receptor, triggering the release of the TM fusion peptide (FP) and its insertion into the target cell membrane. A receptor-induced rearrangement in SU then promotes a second interaction between an RBD and the C-terminal domain of SU, promoting isomerization of the intersubunit disulfide bond, dissociation of SU and TM, and transition of TM from its extended form to the six-helix bundle associated with completion of fusion [120,182]. Isomerization is followed by fusion of the viral and cellular membranes. The experimental observations that sRBDs can rescue fusion in trans, together with direct and indirect evidence for cross-talk, raises the possibility that these steps could involve interactions between protomers of the same trimer or between adjacent trimers [177,183,184] Interestingly, an Env chimera comprising the N-terminal domain and PRR from HTLV-1 (a deltaretrovirus) and the C-terminal domain of SU and all of TM from MLV can mediate superinfection-interference specific for HTLV-1 and is functional for entry [107]. If post-binding interactions between the N- and C-termini are required to complete fusion, this result suggests that the mechanism may also extend to deltaretroviruses.

### 4.2. Gamma-Type Env Preferentially Use SLC Superfamily Transporters as Entry Receptors

A striking property of gamma-type Envs is a significant bias towards use of multi-membrane spanning transporters as entry receptors [7,8,12]. Indeed, a majority of the receptors that have been identified for gammaretroviruses and deltaretroviruses fall into the solute carrier (SLC) superfamily of cellular transporters (Table 1). This is in contrast to viruses in the *betaretrovirus*, *epsilonretrovirus* and *lentivirus* genera, for which no discernible, genus-specific patterns have emerged with respect to receptor preferences [8].

While alpharetroviruses encode a gamma-type Env, a majority do not use SLC receptors; a notable exception is ALV-J, which uses avian Slc9a1 [196]. This distinction between alpharetroviruses and the gammaretroviruses and deltaretroviruses is intriguing. One possibility is that SLC preference is an ancestral property of gamma-type Env, and that this bias was lost during the early evolution of the alpharetrovirus lineage, allowing access to a broader range of potential receptor-specificities. Experimentally, ALV Envs could provide a useful comparator for exploring possible explanations for the SLC preference exhibit by the gammaretroviruses and deltaretroviruses.

A majority of the mammalian syncytins described are gamma-type Env proteins and with one exception, the known syncytin receptors belong to the SLC superfamily [50,214,221,222]. The exception is mouse Syncytin-A, which uses a glycosylphosphatidylinositol (GPI)-anchored membrane protein, lymphocyte antigen 6E (Ly6e), as a receptor [221]. Thus, murine Syncytin-A provides another opportunity to employ comparative approaches to explore the underlying basis for the observed bias of gammaretroviral Envs for SLC receptors.

The gamma-type Env receptors identified thus far include 10 divergent SLC families, including examples with distinct structural folds and different numbers of membrane spanning segments (Table 1). Several gammaretroviruses, and even some retroviruses of divergent genera, have been found to share the same SLC receptors [7]. Additionally, it is common for these viruses to bind to closely related receptors as well, of which there are many within the SLC superfamily. Some viruses are able to use multiple receptors within the same subfamily. For example, gibbon ape leukemia virus (GALV) and feline leukemia virus B (FeLV-B) can both utilize slc20a1 (PiT1) and slc20a2 (PiT2), though slc20a1 is the primary receptor. FeLV-B can also bind to both the feline and human versions of these receptors [7,12]. Thus, receptor-switching may not necessarily be limited to related receptors of the same SLC family. Experimental screening of FeLV based libraries containing randomized sequences in the RBD on a nonpermissive cell line (i.e., receptor-null) identified an Env mutant that could use a unique SLC receptor, SLC35F2, and another mutant that could use human SLC52A2 [208,211,223,224]. SLC52A2 is also used by porcine endogenous retrovirus PERV-A, whereas SLC35F2 is unrelated to any of the previously identified FeLV receptors, and in fact, belongs to a different SLC class and has a different number of membrane-spanning domains (Table 1). Together, these observations suggest that the gammaretroviral Env structure is optimized for use of SLC receptors in a way that is independent of the sequence-specific binding interactions that define the unique host/tissue tropism characteristics of individual viruses.

Given the diversity of SLCs used by viruses with gamma-type Envs, it is highly improbable that this bias reflects a common binding site sequence or receptor motif, and there are no obvious sequence similarities between the mapped viral binding sites recognized by different gammaretroviruses using different SLC receptors. Instead, the receptor preferences of gamma-type Envs may involve two levels of specificity. One level is represented by sequence-specific binding unique to each virus-receptor pair, while another level reflects dependence on one or more sequence-independent features shared by otherwise highly divergent SLC transporter proteins. The former reflects each virus’s association with a specific SLC, while the latter suggests a general dependence on the distinctive architecture of SLC proteins for optimal viral entry. How SLC architecture is involved (and at which step in the entry process) is unclear but represents an intriguing avenue for future exploration.

### 4.3. Recent Structural Insights into the SU-Receptor Interaction

High resolution structural insights into the gamma-type Env include crystal structures of at least three unbound RBDs (Figure 4), as well as papers describing structures of a PRR and the post-fusion conformation of TM [23,108,109,112,225,226,227]. There are also at least two cryo-EM reconstructions of gammaretroviral Envs bound to their cognate receptors [117,228]. Riedel et al. reported low-resolution reconstructions of the MLV trimer, including a receptor-bound form [117]. The trimer roughly resembles a three-sided pyramid, with a base comprising three widely spaced SU C-terminal domains, “steps” consisting of the three PRRs, and an apex consisting of the three RBDs. The authors speculate that the receptor-bound structure represents an intermediate step in entry, with the RBD bound to receptor and TM in its extended conformation linking the viral and cell membranes.

A high-resolution Cryo-EM-derived structure of a syncytin in complex with its cognate receptor has also been described [228]. The Env was derived from a consensus of homologs of Syncytin-2 (SYNC2), one of the two major, placental syncytins of humans and related primates [26]. The receptor for SYNC2 is MFSD2A (SLC59A1) [214]. Micrograph image data included evidence that a single SYNC2 trimer can interact with up to three receptor molecules, with the caveat that this could be a consequence of the experimental procedure which relies on presentation of SYNC2 and MFSD2A on individual micelles [228]. While prior high-resolution structures were limited to the N-terminal RBD of gammaretroviral SU domains, the SYNC2-MFSD2A complex includes high-resolution detail of the RBD as well as most of the C-terminal half of SU. To prevent isomerization and dissociation of SU and TM, the first C in the CXXC motif of the SYNC2 consensus was substituted with an S. Thus, the SYNC2-MFSD2A complex structure may also represent an intermediate receptor-bound step in which TM is still in its extended conformation (and still covalently linked to SU). Unfortunately, the first ~45 residues of SU and all of TM were not modeled in the structure and, in contrast to MLV, the CXXC motif in SYNC2 SU is N-terminal to the RBD at positions 43–46. Thus, the location of the intersubunit disulfide bond during this stage of entry was not revealed by the SYNC2-MFSD2A structure. 

One face of the reported SYNC2 SU structure is largely devoid of N-glycans, and the authors suggest that this represents the SU-TM interacting surface [228]. If so, this places the proline-rich region (PRR), which acts as a hinge connecting the N- and C-terminal domains of SU, on the opposite side of the protomer from the extended TM. This placement is also consistent with the MLV structure reported by Riedel et al., with the PRR oriented towards the outside of the trimer [117]. 

The structure of SYNC2 SU reveals two domains: a beta-barrel C-terminal core, and an N-terminal RBD consisting of a five-stranded beta sheet and an alpha-helix, connected by the PRR [228]. The RBD includes the three variable regions (referred to by the authors as receptor-binding loops or RBLs 1–3). The interface between SYNC2 and MFSD2A involves extensive contacts between the variable loops and interstices formed by multiple extracellular loops (ECLs) of the receptor. This places the tips of the variable loops very close to the surface of the membrane, including one that penetrates into the plane of the target membrane and puts a phenylalanine (F275) in possible contact with lipid. Importantly, several features of the structure are consistent with mutagenesis and functional assays, including those conducted by the authors and those reported in the literature [118,228,229]. It is tempting to speculate that the penetration of the SYNC2 RBLs close to or within the plane of the target membrane could reflect a common feature of gamma-type Envs that use SLC receptors, and that this may help to explain the observed bias towards SLC superfamily members as entry receptors.

Human Synctyin-1 (SYNC-1) is homologous to the ENV proteins of retroviruses in the RDR receptor interference group, including the simian type-D betaretroviruses. All RDR Env tested to date, including SYNC1, use homologs of ASCT2/ASCT1 (SLC1a5, SLC1a4) as receptors [6]. In addition to extant exogenous retroviruses, the RDR Env is found in a diverse array of ERV families found in the genomes of various mammalian species, including human HERV-W loci (and SYNC1 is encoded by a HERV-W *env* gene). RDR represents a group of retroviruses that share a homologous Env protein, very likely as a consequence of numerous recombination events between different retroviral lineages taking place over a span of tens of millions of years [3,6]. Recently published Alphafold predictions suggest that the RDR RBDs may adopt a fold distinct from those of MLV, FeLV and EnvP(b)1 [230], such that current structures may not be suitable for gaining insights into this important class of gamma-type Env, including human SYNC1.

### 4.4. Receptor Interference

**Figure 6:**
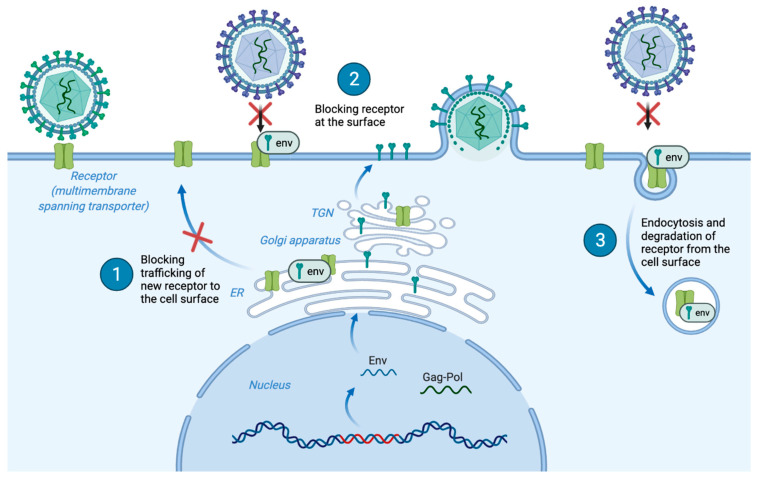
Receptor Interference (Superinfection Resistance). Env expression in infected, virus-producing cells induces receptor interference. Proposed mechanisms include: (1) blocking trafficking of the receptor to the cell surface; (2) binding of the receptor at the cell surface, thereby blocking new viral particles from binding, and (3) inducing endocytosis and subsequent degradation of the receptor.

Actively infected cells often exhibit resistance to further infection from retroviruses that utilize the same receptor, a phenomenon commonly referred to as receptor interference or superinfection resistance (Figure 6). This property of retroviruses can be used to group viruses based on pairwise comparisons of interference patterns even when the cognate receptors are unknown [2,100,185,231,232,233,234,235]. For HIV, superinfection resistance involves multiple proteins, including Env and the accessory proteins Vpu and Nef. Vpu prevents trafficking of new CD4 receptors to the cell surface, while Nef causes endocytosis of cell surface CD4 [236]. Retroviruses with gamma-type Env typically have simple genomes with few if any apparent accessory genes (except the deltaretroviruses). Of the few that have been studied (e.g., MLV *glycogen*, HTLV *HBZ*) none have yet been shown to mediate receptor interference. Instead, receptor-interference is governed predominantly or exclusively by Env. 

Previous studies have shown that expression of the murine leukemia virus (MLV) Env is correlated with an internalization of the receptor such that it is distributed throughout the cytoplasm, ER, Golgi and lysosomes, and no longer found on the cell surface [237,238]. Internalization and subsequent degradation have also been demonstrated for a human endogenous retrovirus (HERV) Env [72]. Expression of these Envs, while they are not associated with any active exogenous virus, causes internalization and overall reduction of the receptor, as shown through immunofluorescent imaging experiments and Western blot, respectively [72]. Another study altered Env through addition of ligand sequences into the RBD and showed that this chimeric Env was able to induce strong resistance to superinfection and downregulate its new receptor from the cell surface [239]. Interestingly, previous studies using reticuloendotheliosis virus (REV) have shown that the envelope protein of this virus can induce resistance without being properly trafficked to the cell surface and does not need to be fusogenic to induce resistance, indicating that the regions of the TM subunit of Env involved in fusion are likely not required for inducing resistance to superinfection [96,240]. 

Additionally, multiple endogenous Envs that are no longer capable of fusion are still capable of inducing resistance to further infection, and likely have been co-opted for this purpose, as in the case of murine Fv4 [55]. Multiple point mutations to the RBD of the SNV Env abolished the envelope protein’s ability to induce resistance to superinfection in the cell [96]. On the other side of the Env-receptor coin, mutations to the receptor that reduce binding affinity of the envelope protein also reduce or abolish superinfection resistance [241]. Gibbon ape leukemia virus (GALV) can induce superinfection resistance in a cell without causing any change in density of the receptor at the cell surface; however, it is possible GALV uses two receptors, which may account for the lack of change in the receptor measured [242,243]. 

The commonality of the receptor-interference phenomenon, and the variety of mechanisms employed, implies that inducing cellular resistance to superinfection reflects a benefit to viral fitness. In multiple retroviruses it has been shown that a loss of superinfection resistance is strongly correlated with higher cytotoxicity and abnormal pathogenesis [104,244,245,246,247,248]. As retroviruses rely on their cellular host to produce copies of their genomes and proteins, they depend on the cell’s continued survival, at least long enough to produce progeny. Another possible benefit for the virus may be involved in clearing a path for new envelope proteins trafficking to the cell surface or sites of assembly. Downregulation of receptors would prevent these Envs from prematurely binding and could prevent them from losing fusogenicity before they have been incorporated into virions. 

Based on these and other studies, proposed mechanisms for receptor-interference by gamma-type Env typically fall into one of three general categories (Figure 6). Briefly, these are: (1) Env prevents trafficking of new receptors to the surface, resulting in overall reduction of cell-surface receptor levels; (2) Env binds to the receptor at the cell surface, blocking a subsequent viral particle from binding to that receptor, referred to as receptor masking, and (3) Env promotes downregulation of the receptor from the cell surface through induction of endocytosis. Regardless of mechanism, it is clear that the receptor-recognition function of SU is necessary for receptor interference. It is likely that retroviruses with gamma-type Env differ in which of these mechanisms (or combinations of these mechanisms) are employed to induce superinfection resistance. Further work is needed to uncover the molecular interactions underlying each mechanism, including identification of other regions of Env that contribute and the cellular co-factors and trafficking pathways that are involved. 

## 5. Summary and Outlook

The biological and temporal distribution of the gamma-type Envs in nature is remarkable. They are represented by at least four of the seven genera comprising the *Orthoretrovirinae* subfamily (five, if one includes the type-D betaretroviruses), and they have been acquired by RNA viruses in two other families and at least one large DNA virus [3,14,15,16,17,18,137,230]. The origins of the gamma-type Env are obscure, but likely date back hundreds of millions of years and span most of vertebrate evolution. Gamma-type *env* genes are also present in thousands of copies in most vertebrate genomes, including many instances in which coding capacity has been preserved and exapted for important host functions (e.g., human SYNCYTIN-1 and SYNCYTIN-2) [4,28,38,43]. Thanks to a combination of distinctive features, the gamma-type Env is easily identified, and readily distinguished from the Env proteins of other retroviruses and from other class I fusogens [3,4]. Comparative studies of the gamma-type Env can illuminate additional aspects of viral entry, provide insights into intraspecies and interspecies transmission and emergence of viruses, improve our understanding of Syncytin function (and by extension mammalian evolution), and serve as models for understanding how functional constraints influence evolution of protein structure and protein-protein interactions over deep evolutionary timescales. Among the many possibilities for further exploration, the following represent some of the most significant gaps in current knowledge.

### 5.1. Structural Insights into the Distinctive Entry and Fusion Mechanisms of Gamma-Type Env

While HIV Env has emerged as a model for retroviral entry, the gamma-type Env has several distinctive features that are absent from HIV Env, including regulation by a C-terminal R-peptide, a labile intersubunit disulfide bond, and involvement of the RBD at two steps in the entry process. Understanding these features and how they interact to perform the various functions of the gamma-type Env will necessarily require additional structural insights drawn directly from comparative work involving viruses with gamma-type Env, such as ALV, MLV and HTLV. Work on the structural biology of these viruses lags far behind HIV-1, and in particular, structural details of the immature precursor (with an R-peptide), the mature trimer, and the intermediate stages of fusion are still lacking. Moreover, given the very broad range of viruses that encode gamma-type Env (and the correspondingly broad host distribution), it is likely that novel folds have evolved, particularly in the N-terminal domain of SU. Some SU proteins have an N-terminal CXXC, for example, which may reflect differences in the arrangement or structure of SU and its interface with TM. One of these is SYNC2, and it is noteworthy that the RBD portion of the published SU structure differs significantly from the reported structures of the MLV, FeLV and Envp(b)1 RBDs, all of which represent Env with a CXXC motif in the C-terminal half of SU [23,108,109,228]. The ALVs lack a CXXC, which could also be reflected in differences in conformation of SU and the ALV trimer. Finally, based on alphafold predictions, Hötzel has proposed that distinctive structures may exist for viruses of the RDR group and human Syncytin-1 [230].

### 5.2. Conservation of the ISD Motif

The function(s) of the ISD motif need to be further defined, and a satisfying explanation for the extraordinary conservation of the ISD sequence, across multiple genera and hundreds of millions of years of viral evolution, is lacking. Whether the proposed immunosuppressive function alone is sufficient to explain this conservation will depend on whether immunosuppression is shared across divergent retroviruses representing the *alpharetrovirus*, *gammaretrovirus* and *deltaretrovirus* genera, and a correspondingly wide range of vertebrate hosts, and on identifying a plausible molecular mechanism that unifies the various published observations. Alternatively, or possibly in addition to immunosuppression, it is possible that conservation of the ISD sequence belies a critical contribution to the essential entry-related functions shared by all gamma-type Envs.

### 5.3. Receptor Bias

There is as yet no molecular explanation for the observed bias of gammaretroviruses and deltaretroviruses towards use of SLC transporters as receptors. Solving this puzzle may depend on obtaining additional high-resolution structures or robust structural models of Env trimers in various stages of the entry process. The recent high-resolution structure of SYNC2 in complex with MSFD2A revealed the insertion of variable domains deep into the receptor, close to or within the plane of the target membrane. If this tight junction between the RBD and the cell is shared by other Env-receptor pairs and is optimal for a subsequent step in the entry process, this may begin to explain the bias towards the low-profile receptors typical of the SLC superfamily. The technical challenges of experimenting with complex, multi-functional viral glycoproteins and multi-membrane spanning receptors are formidable. In addition, confirming fundamental hypotheses for SLC receptor preference will require expanding on traditional single-virus model systems to include broader, comparative approaches involving parallel analysis of divergent gammaretroviruses with different receptor specificities in order to identify common themes. Some clues may also come from a closer examination of the “exceptions to the rule”—these include avian leukemia virus subgroups A-E, which do not use SLCs as receptors (except for ALV subtype J [196]), and murine Syncytin-A, which uses the GPI-anchored Ly6e protein as a receptor [221].

### 5.4. Virus-Host “Arms Races” and the Evolution of Individual SLC Receptors

There are a few studies that have examined the potential impact of gammretroviruses on the evolution of the genes that encode their cognate receptors [249,250,251]. There are now several examples of SLC receptors that are used by multiple retroviruses (Table 1), as well as cases where the receptors used in the deep past by ancient ERV Env have been identified [50,72,188,206,214,221,222]. Testing the Env proteins of these viruses against an expanded array of receptor homologs, coupled with molecular evolutionary analysis of the corresponding host genes, could be used to gauge the impact of viruses on vertebrate evolution generally and receptor evolution specifically.

### 5.5. The Link between Entry and Post-Entry

The ubiquity of gamma-type ENVs and the diversity of receptors involved is a unique opportunity to understand how Env-receptor interactions dictate both entry pathways and subsequent trafficking of the capsid core (e.g., [252,253]).

### 5.6. Env Trafficking and Receptor-Interference

While it is well-established that gammaretrovirus Env proteins achieve superinfection resistance through receptor-interference, detailed mechanisms remain to be identified, and alternative hypotheses have been proposed (and it is possible that different viruses have evolved distinct mechanisms to achieve receptor-interference). Recently, Eksmond et al. revisited an MLV Env ISD mutant and uncovered a receptor-dependent, producer cell effect on infectivity [147]. Given that superinfection resistance is also a receptor-dependent, producer-cell effect mediated by Env, their observations could be related to the underlying mechanism of receptor interference. More detailed examination of Env biogenesis and trafficking in the presence and absence of cognate receptors, and the identification of any cellular cofactors involved, may also shed light on the superinfection process and its relevance to viral replicative fitness, virion release and cell-cell spread [242].

### 5.7. Evolutionary Cooption of ERV-Encoded Gamma-Type Env Proteins

Because their origins and underlying virological functions are well understood, ERV-encoded Env glycoproteins can serve as model systems for understanding the phenomenon of evolutionary cooption and the events that occur after germline integration of an *env* gene, such as acquisition and fine-tuning of host-cell regulatory mechanisms, integration within the regulatory networks of other interacting host genes, and further adaptations of the encoded Env protein for optimal host function [25,27]. For example, decades of work on exogenous viruses such as MLV-guided identification of the fusion defects in the murine Fv4 and human envHERV-T viral resistance proteins [66,69,72]). Syncytins, by contrast, retain their fusogenic activity, and do so in the absence of activation by a viral protease. This suggests that Syncytins must either acquire alternative activation mechanisms, or evolve to function independently of R-peptide cleavage [254]. Aside from work performed in experimental mouse models [255,256], additional research is also needed to confirm and further define the presumed function(s) of many of the placental syncytins of other mammalian lineages. Finally, there are many ERV gamma-type Env “orphans” in the genomes of humans and other vertebrates [27,45,46], including the human HEMO and EnvP(b)1 proteins [22,23,48] and the Percomorf protein of ray-finned fish [24], for which host functions remain to be identified.

## Figures and Tables

**Table 1:** Known receptors used by viruses with gamma-type Envs for cell entry.

Receptor	Transporter of	Fold	# of Membrane-Spanning Domains	Virus	Classification	Source
Slc1a4 (ASCT1)	Neutral AA	Glt	8	REV	Gammaretrovirus	[185]
Slc1a4 (ASCT1)	Neutral AA	Glt	8	RD114	Gammaretrovirus	[186]
Slc1a4 (ASCT1)	Neutral AA	Glt	8	SNV	Gammaretrovirus	[187]
Slc1a4/Slc1a5 (ASCT1/ASCT2)	Neutral AA	Glt	8	HERV-W/SYNCYTIN-1	Gamma-like ERV	[50]
Slc1a5 (ASCT2)	Neutral AA	Glt	8	SUPYN	Gamma-like ERV	[188]
Slc1a5 (ASCT2)	Neutral AA	Glt	8	SRV1, SRV2, SRV3 (MPMV), SRV4, SRV5	Betaretrovirus *	[189]
Slc1a5 (ASCT2)	Neutral AA	Glt	8	BaERV	Gammaretrovirus	[190]
Slc2A1 (Glut-1)	Glucose	MFS	12	HTLV	Deltaretrovirus	[191]
Slc5a3 (Smit1)	Sodium/myo-inositol	LeuT	12	M813	Gammaretrovirus	[192]
Slc5a3 (Smit1)	Sodium/myo-inositol	LeuT	12	HEMV	Gammaretrovirus	[193]
Slc7a1 (mCAT1)	Cationic AA	LeuT	12	MoMLV	Gammaretrovirus	[194]
Slc7a1 (CAT1)	Cationic AA	LeuT	12	BLV	Deltaretrovirus	[195]
Slc9a1 (NHE-1)	Sodium/Hydrogen	NHE1	13	ALV-J	Alpharetrovirus	[196]
Slc16a1 (MCT1)	monocarboxylate	MFS	12	HERV-T	Gammaretrovirus	[72]
Slc19a1	Folate	MFS	12	GLN-MLV	Gammaretrovirus	[197]
Slc19a2 (THTR1)	Thiamine	MFS	12	KoRV-B	Gammaretrovirus	[198]
Slc19a2 (THTR1)	Thiamine	MFS	12	KoRV-J	Gammaretrovirus	[199]
Slc19a2 (THTR1)	Thiamine	MFS	12	FeLV-A	Gammaretrovirus	[200]
Slc20a1 (PiT1)	Phosphate	PiT	10, 12	KoRV-A	Gammaretrovirus	[201]
Slc20a1 (PiT1)	Phosphate	PiT	10, 12	FeLV-B	Gammaretrovirus	[202]
Slc20a1/Slc20a2 (PiT1/PiT2)	Phosphate	PiT	10, 12	GALV/WMV	Gammaretrovirus	[203]
Slc20a1 (PiT1)	Phosphate	PiT	10, 12	AMLV (4070A, 229A)	Gammaretrovirus	[204]
Slc20a1 (PiT1)	Phosphate	PiT	10, 12	FeLV-T	Gammaretrovirus	[179]
Slc20a1 (PiT1)	Phosphate	PiT	10, 12	10A1-MLV	Gammaretrovirus	[205]
Slc31a1 (CTR1)	Copper ion	Ctr	3	cERV-1/2	Gamma-like ERV	[206]
Slc31a1 (CTR1)	Copper ion	Ctr	3	FeLV-D	Gammaretrovirus	[207]
Slc35f2	Nucleotide sugar	-	10	FeLV-A5 *	Gammaretrovirus	[208]
Slc49a1/Slc49a2	Heme	MFS	12	FeLV-C	Gammaretrovirus	[209]
Slc52a2 (GHB/RFVT2/ PAR-2)	Riboflavin	MFS	12	PERV-A, FeLV-CP *	Gammaretrovirus	[210,211]
Slc53a1 (Xpr1)	Phosphate	Slc53	8	XMLV	Gammaretrovirus	[212,213]
Slc53a1 (Xpr1)	Phosphate	Slc53	8	PMLV	Gammaretrovirus	[212,213]
Slc59a1 (MFSD2A)	Lysophosphatidylcholine	MFS	12	HERV FRD/SYNCYTIN-2	Gamma-like ERV	[214]
Slc65a1 (NPC1)	Cholesterol	NPC1	13	Ebola virus	Non-retrovirus (filovirus)	[215]
TVA	Cobalamin (?)	LDLR	1	ALV-A/RSV-A	Alpharetrovirus	[216,217]
CAR1 (TVB)	Tumor necrosis factor	TNFR	1	ALV-B/D/E	Alpharetrovirus	[218]
TVC	Immunoglobulin	Ig	1	ALV-C	Alpharetrovirus	[219]

Receptors are listed with their SLC classification and commonly used name, if applicable. Fold refers to classification used in [220]. The “*” indicates artificial isolate selected from a library screen. Slc = designates member of the solute carrier superfamily. ASCT1/2 = Alanine Serine Cysteine Transporter type 1/2; Glut-1 = Glucose transporter protein type 1; Smit-1 = Sodium myo-inositol transporter type 1; mCAT1 = murine cationic amino acid transporter type 1; CAT1 = cationic amino acid transporter type 1; NHE-1 = sodium hydrogen exchanger type 1; MCT1 = Monocarboxylate transporter type 1; THTR1 = Thiamine transporter 1; PiT1 = Phosphate transporter; CTR1 = Copper uptake protein 1; GHB = gamma-hydroxybutyrate receptor; RFVT2 = Riboflavin uptake transporter; PAR2 = Protease activated receptor; Xpr1 = xenotropic and polytropic retrovirus receptor 1; MFSD2 = Major facilitator superfamily domain-containing protein 2; NPC1 = Niemann-Pick type C1 cholesterol transporter; TVA/B/C = tumor virus A/B/C.

## Data Availability

No new data were created for this review article.
